# Editorial: Novel Targets and Targeting Technologies to Modulate Tumor Microenvironment

**DOI:** 10.3389/fonc.2016.00121

**Published:** 2016-05-17

**Authors:** Jai Prakash, Kristian Pietras

**Affiliations:** ^1^Targeted Therapeutics, Biomaterials, Science and Technology, MIRA Institute for Biomedical Technology and Technical Medicine, University of Twente, Enschede, Netherlands; ^2^Department of Laboratory Medicine, Division of Translational Cancer Research, Lund University, Lund, Sweden

**Keywords:** tumor microenvironment, tumor stroma, microRNA, targeted therapeutics, cancer-associated fibroblasts, stromal cells

**The Editorial on the Research Topic**

**Novel Targets and Targeting Technologies to Modulate Tumor Microenvironment**

In recent years, there is an increasing attention on the impact of the tumor microenvironment (TME) on tumor initiation, progression, and metastasis (1). Malignant tumor cells and non-malignant stromal cells actively interact with each other and thereby create a tumor-supportive microenvironment (2). TME is composed of extracellular matrix and several cell types, such as cancer-associated fibroblasts (CAFs), endothelial cells, pericytes, tumor-associated macrophages (TAMs), and other immune cells (1, 3). The literature has been continuously highlighting new aspects on the complexity of these cell types and their pro- and anti-tumorigenic roles in cancer. For instance, depletion of stroma in pancreatic cancer has been shown to induce tumor growth (4), in contrast to literature showing tumor-promoting effects of stroma (2). There is a continuous increase in revelations of novel cellular and extracellular targets in TME, such as new receptors, extracellular matrix, and microRNA (miRNAs). Modulation of these targets may lead to the development of new diagnostics and therapeutics for cancer. To reinforce these developments, it is crucial to involve targeting technologies, such as drug and gene-targeting technologies, to TME.

Ehnman and Larsson have reviewed the microenvironmental targets in sarcomas, which are rare malignant tumors in all age groups. Treatment of sarcoma has faced several failures in clinical trials, urging a need for developing novel targeted therapies in combination with current multimodality regimens. Vasculature cells (e.g., endothelial cells, pericytes), immune cells (e.g., M2-like macrophages, lymphocytes), and fibroblast-like cells are the crucial stromal cells in sarcomas. In clinic, therapies have been focused on inhibiting angiogenesis (e.g., tyrosine kinase inhibitors, including VEGFRs, PDGFRs, and c-KIT) and immune modulation (e.g., monoclonal antibodies against RANK receptor). Several therapies that are under investigation focus on the inhibition of multiple kinases simultaneously.

Kuninty et al. highlight miRNAs as key therapeutic targets in TME. miRNAs, small endogenous non-protein-coding RNAs, are emerging as key molecules in the modulation of TME due to their capacity to control cellular processes by inhibiting the expression of multiple target genes. The review enlists miRNAs dysregulated in different stromal cells, including CAFs, TAMs, immune cells, and tumor vascular cells (endothelial cells, pericytes). Due to the inherent physicochemical properties of miRNA (negative charge and hydrophilicity), they cannot pass through the cell membrane. Therefore, novel miRNA delivery systems are crucial to develop in order to target miRNA/anti-miRNA into stromal cells. Delivery strategies based on non-viral delivery systems, such as liposomes, lipoplexes, and polyplexes, are the state-of-the-art carriers to deliver miRNA.

Elkhattouti et al. have focused on the role of stromal fibroblasts in tumorigenesis in age-related cancer. With advanced age, stroma generates a pro-tumorigenic microenvironment composed of cytokines, chemokines, and high energy metabolites. These factors drive the age-related cancer initiation and maintain its progression. With ageing of stroma, stromal fibroblasts become senescent so-called senescence-associated secretory phenotype (SASP) and contribute to tumor progression. Local stromal delivery of IL-12, an antitumor cytokine targeting immunosuppressive mechanisms, and intervention into the processes, such as fibroblast-fueled metabolites to cancer cells, are proposed as vital strategies to inhibit stromal tumorigenic activities.

Venning et al. have highlighted the role of ECM in different processes of metastatic spread and benefits of targeting ECM to prevent cancer progression. Key ECM players, such as periostin, tenascin C, hyaluronan, versican, and collagen-I are involved in the metastatic process. Many ECM proteins are involved in the induction of EMT in tumor cells by activating their signaling and helping them escape the primary tumor to blood. Within the blood, the ECM protein fibrinogen forms complexes with platelets and protects tumor cells from immune surveillance. Furthermore, at the metastatic site, ECM deposition and remodeling facilitates the outgrowth of metastasis. Targeting ECM proteins specifically using antibodies, peptides, or aptamers have been shown to target and block metastasis formation.

Most tumors are abnormal in the structure of the tumor vasculature and stroma. Gkretsi et al. highlight the remodeling TME to improve cancer therapy. Abnormal tumor vasculature, as a result of angiogenesis and ECM deposition, severely affects the accumulation of chemotherapies into tumors. Several strategies to remodel TME have been proposed, including vascular normalization using anti-VEGF therapies to improve drug delivery and stress alleviation using angiotensin receptor blockers to reduce intratumoral pressure. The latter approaches have been shown to improve the effect of chemotherapies and nanomedicines in preclinical models.

Boonstra et al. have proposed an interesting approach to utilize stroma targeting for image-guided surgery. Image-guided oncologic surgery (IGOS) assists the surgeon to distinguish tumor tissue from normal tissue during operation. Currently, fluorescent dyes in the near-infrared range are used in clinic, either in free form or conjugated to a ligand (antibody, peptide) against targets present on tumor vasculature (αvβ3) or tumor cells (targets: EGFR, EpCAM). However, stromal cells as targets are not commonly used for IGOS. Targeting to CAFs and macrophages can be an attractive possibility to identify stroma as a margin and use for surgical removal of the whole tumor mass.

The research article by Yan et al. show the design of a cancer-selective peptide and its application as a hybrid peptide PTS for rapid imaging of tumors preclinically. A hybrid peptide consisting of a cell-penetrating peptide and αvβ3-binding peptide was synthesized and labeled with near-infrared dye. The hybrid peptide showed more cellular uptake *in vitro* compared to individual peptides. *In vivo* using NIR imaging, the hybrid peptide was shown to be accumulated into tumors to a greater extent compared to the individual peptides. This approach shows the applicability of using combined targeting peptides for molecular imaging that could be translated into clinics in future.

In conclusion, this special issue highlights the key elements of TME; in particular, stromal targets such as miRNA, kinase pathways, and ECM proteins are highlighted to design novel therapeutics against cancer. In addition, targeting technologies to deliver miRNA as therapeutics and imaging probes for molecular imaging and image-guided surgery directing to stroma are also highlighted in this review issue. In future, integration of stromal targets with targeting technologies will pave new ways to utilize stroma for tumor diagnosis, surgery, and treatment.

## Author Contributions

The author JP wrote the editorial, and the author KP edited it.

## Conflict of Interest Statement

The authors declare that the research was conducted in the absence of any commercial or financial relationships that could be construed as a potential conflict of interest.
